# Exercise improves subchondral bone microenvironment through regulating bone-cartilage crosstalk

**DOI:** 10.3389/fendo.2023.1159393

**Published:** 2023-05-23

**Authors:** Shihua Zhang, Tingting Li, Yao Feng, Keping Zhang, Jun Zou, Xiquan Weng, Yu Yuan, Lan Zhang

**Affiliations:** ^1^ School of Exercise and Health, Shanghai University of Sport, Shanghai, China; ^2^ School of Exercise and Health, Guangzhou Sport University, Guangzhou, China; ^3^ College of Sports and Health, Shandong Sport University, Jinan, China

**Keywords:** bone, exercise, bone-cartilage crosstalk, osteoarthritis, mechanical stress

## Abstract

Articular cartilage degeneration has been proved to cause a variety of joint diseases, among which osteoarthritis is the most typical. Osteoarthritis is characterized by articular cartilage degeneration and persistent pain, which affects the quality of life of patients as well as brings a heavy burden to society. The occurrence and development of osteoarthritis is related to the disorder of the subchondral bone microenvironment. Appropriate exercise can improve the subchondral bone microenvironment, thus playing an essential role in preventing and treating osteoarthritis. However, the exact mechanism whereby exercise improves the subchondral bone microenvironment remains unclear. There is biomechanical interaction as well as biochemical crosstalk between bone and cartilage. And the crosstalk between bone and cartilage is the key to bone-cartilage homeostasis maintenance. From the perspective of biomechanical and biochemical crosstalk between bone and cartilage, this paper reviews the effects of exercise-mediated bone-cartilage crosstalk on the subchondral bone microenvironment, aiming to provide a theoretical basis for the prevention and treatment of degenerative bone diseases.

## 1 Introduction

Osteoarthritis (OA) may be influenced by genetic, environmental, metabolic, and biochemical factors. However, the majority of the time, cartilage wear brought on by aberrant mechanical forces in the joint results in OA (1). OA patients frequently have joint dysfunction due to pain and the deterioration of their joint cartilage, which has a negative impact on their quality of life. Currently, there are both surgical and non-surgical options for treating OA. The main surgical treatments include arthroplasty, and non-surgical treatments include medication and exercise therapy. Exercise therapy is crucial to non-surgical OA treatment since it is efficient, practical, and affordable. It is also adjustable, requires little location or corresponding equipment, and has no side effects when used consistently over time (1). Improving osteoarthritis treatment options is important in relieving symptoms, improving patients’ quality of life, and maximizing social and financial advantages. Currently, numerous studies have confirmed that exercise can prevent and treat osteoarthritis (2–4). A proper amount of aerobic exercise can slow the progression of OA and lessen the patient’s symptoms (5). On the contrary, overtraining can lead to mechanical overloading of weight-bearing joints, which induces degeneration of articular cartilage and eventually OA lesions (6, 7). When considered collectively, the existing research on how exercise affects osteoarthritis suggests that any favorable or unfavorable changes depend on mechanical stress on the subchondral bone transition. This review investigates the role mechanical stress stimulation plays in transmitting biochemical and biomechanical signals between these cells or tissues and considers whether this role might offer the possibility of therapeutic interventions to halt or slow disease progression.

## 2 The subchondral bone microenvironment in osteoarthritis

### 2.1 Structural characteristics of the subchondral bone microenvironment in osteoarthritis

Its pathological changes are characterized by focal destruction of articular cartilage within the synovial joint, accompanied by subchondral bone sclerosis and bone redundancy (8). Studies have shown that (9) osteoarthritis is influenced by various factors such as age, genetics, obesity, hormones, and trauma. If these risk factors are not taken seriously, patients may experience joint pain, dysfunction. As the disease progresses, it can potentially cause disability and some psychiatric or psychological difficulties. OA is now the leading cause of pain, limited range of motion, and loss of joint motion in older adults (10), which seriously affects the health of life and quality of life.

Additionally, OA as a widespread debilitating disease will place a significant financial burden on patients and society due to global aging, obesity, and increased joint injury (11). Normal wear and tear, abnormal mechanical loading, injury, and aging are common causes of damage to articular cartilage as well as subchondral bone, synovial tissue, and ligaments, which may alter the molecular composition and organization in the extracellular matrix. Under stimulation, injured chondrocytes produce matrix metalloproteinases (MMP-1, MMP-3, and MMP-13) and ADAMTS (ADAMTS-4 and ADAMTS-5). They lead to a decrease in the levels of proteoglycans, aggregated glycans, and type II collagen in the cartilage matrix by inhibiting the synthesis of critical components of the extracellular matrix, ultimately leading to cartilage degeneration (12). According to studies, the subchondral bone mineral density (BMD), trabecular volume fraction (BV/TV), trabecular number (Tb.N), and trabecular thickness (Tb.Th) of the tibial plateau are all positively connected with the severity of osteoarthritis, which was based on cartilage defects and thinning as well as histological score (13), Kellgren-Lawrenc e (K-L) grading (14), and cartilage defects.

Subchondral bone typically refers to the epiphyseal bone area located immediately below the calcified cartilage. And some scholars contend that this concept should be extended to calcified structures distal to the tidal line of articular cartilage, and calcified cartilage should be classified as subchondral bone (15, 16). Anatomically, subchondral bone structures can be further classified as either subchondral bone plates or subchondral bone trabeculae (17). A distinctive area at the bone interface between the articular cartilage and the long bones of the joint consists of articular cartilage underlain by calcified cartilage located within the subchondral bone plate, which in osteoarthritis has marked progressive destructive changes. Firstly, the subchondral bone plate is a thin lamellar structure (17), similar to the cortical bone in other skeletal areas. Moreover, the subchondral plate is also able to connect the articular cartilage directly to the subchondral bone (18, 19), beneath which is the cancellous bone at the end of the long bones, with internal pores that serve as a direct connection between the articular cartilage and the subchondral trabeculae. The density and distribution of these pores are primarily influenced by the degree of osteochondral bone aging and the magnitude of compression forces transmitted within and between joints via the osteochondral bone (20). These channels are preferentially concentrated in high stress areas within the joint, and as the thickness of the subchondral bone plate changes, the shape and diameter of the channels appear to change accordingly: in areas with thicker subchondral bone plates, the channels are narrower and form a mesh, while areas with thinner subchondral bone plates are wider (20).

Compared to the subchondral bone plate, the subchondral trabeculae contain blood vessels, sensory nerves, and bone marrow with lax porosity and more active metabolic activity (21). The trabeculae of the subchondral bone play a clear role in joints without lesions by acting as essential supports and shock absorbers. At the same time, it can promote the cartilage’s metabolic processes and offer essential nutritional support, all of which play a crucial role (15). The knee joint is distinct from the bones and joints of the extremities in that it is covered in cartilage and subchondral bone. The cartilage of the femoral condyles of the knee joint is rich in blood vessels and nerves, and small trabecular branches enter the calcified cartilage layer. The subchondral bone and the articular cartilage are closely linked. When correctly united, the subchondral bone and articular cartilage create the osteochondral junction. This unique functional unit is capable of preserving and stabilizing the homeostasis of the intra-articular environment (22). In fact, numerous potential functions for components like subchondral bone and calcified cartilage in the development of osteoarthritis exist. The majority of current clinical imaging studies, however, are based on calcified subchondral tissue, and current imaging techniques are unable to distinguish anatomically between subchondral bone and calcified cartilage. Additionally, the absence of distinct anatomical boundaries between the various regions of subchondral bone makes it challenging to conduct in-depth research on the bone’s role.

### 2.2 Bone remodeling in osteoarthritis

#### 2.2.1 The role of bone remodeling in osteoarthritis

In recent years, as research on osteoarthritis has intensified, people no longer view osteoarthritis as a condition primarily characterized by articular cartilage degeneration and intermittent joint stenosis but rather as a total joint disease affecting articular cartilage, subchondral bone, synovium, ligaments, meniscus, and periarticular muscles. If untreated, subchondral bone lesions that develop early can become osteoarthritis (23). According to animal studies, in early OA, subchondral bone resorption exhibits a transitory increase in subchondral bone resorption and a decrease in bone volume fraction (24, 25). Early in the onset of osteoarthritis, bone remodeling is enhanced due to an accelerated process that delays the completion of mineralization of osteoid, leading to a predominance of bone resorption. However, when the progression of arthritis intensifies, aberrant bone remodeling reduces the subchondral bone’s capacity to conduct stress, coupled with the altered biomechanical structure of the bone, leads to uneven deformation of the cartilage under stress and shear forces, producing cartilage damage such as cracks (23). Thus it can be seen that the dynamic equilibrium of bone remodeling is an essential factor in the development and progression of arthritic disease.

Bone remodeling proceeds closely to the pathological changes of arthritis in a process that includes four stages: activation, resorption, reverse, and formation. The clear boundary between articular cartilage and calcified cartilage is called the tideline, and the subchondral bone deep in the tideline is connected to the articular cartilage by calcified cartilage. In osteoarthritis, there is enhanced calcification of the deeper articular cartilage, leading to an upward shift of the tide line and subsequent thinning of the articular cartilage (26). The highly vascularized synovial membrane secretes synovial fluid, which feeds articular cartilage, while the deeper chondrocytes receive nutritional support from the subchondral bone (22). Subchondral bone sclerosis is another characteristic change in addition to cartilage loss as OA progresses. In the subchondral bone microarchitecture, this is manifested by a decrease in the number of trabeculae, an increase in thickness, a more dispersed arrangement, and an increase in the bone volume fraction (Bone volume/Tissuevolume, BV/TV) (27). Subchondral bone sclerosis is particularly prominent beneath areas of severe cartilage defects in advanced OA. The subchondral bone plate is considerably thickened, and the degree of cartilage defect is favorably connected with the subchondral bone lesion (13, 28, 29).

#### 2.2.2 Important cytokines involved in bone remodeling in arthritis

Endochondral ossification is the process that supports longitudinal bone growth during skeletal maturation. In healthy individuals, this process begins when mesenchymal cells proliferate, differentiate into pre-chondroblasts, and further differentiate into chondroblasts. Chondroblasts embed into the cartilage matrix they secrete and further differentiate into chondrocytes, forming very early bone prototypes. Chondrocytes primarily produce matrix molecules, but they also secrete growth factors, including the receptor activator of nuclear factor (NF)-ĸB-ligand (RANKL) and vascular endothelial growth factor(VEGF), which promote vascular invasion and osteoclast recruitment (30). Periosteum around the prototype is differentiated from mesenchymal cells, and osteoblasts are beginning to form a layer that resembles bone (31). Mammalian bone tissue is formed during embryonic development by two distinct processes. Intramembranous bone formation produces many craniofacial bones directly from mesenchymal coalescence, while endochondral ossification is the primary process of mammalian bone formation, generating bone through the cartilaginous middle. The transformation of cartilage to bone is closely related to chondrocyte, osteoblast, and vascular differentiation.

During the progressive hypertrophy of chondrocytes, cells within the perichondrium differentiate into osteoblasts, and the perichondrium is further vascularized. Vascular invasion into hypertrophic chondrocytes is a critical step. Blood vessels spread osteoblast precursors and osteoclasts into the cartilage matrix, eroding the cartilage matrix while secreting type I collagen to form cancellous bone. Osteoblasts in the periosteum then secrete a highly calcified matrix that forms cortical bone (32). The process of endochondral bone formation is regulated by the interplay of various hormones, growth factors and signaling pathways. For example, parathyroid hormone-related peptide(PTHrP) (33–36), Indian hedgehog (IHH) (37–39), fibroblast growth factor (FGFs) (40–42), bone morphogenetic protein (BMP) (43, 44), WNTs signaling (45), Notch signaling (46, 47), etc. These factors and their associated signaling pathways interact to activate key transcription factors in osteoblast differentiation through various pathways, including SOX9 (48, 49), Runx2 (50, 51), OSX (52, 53), ATF4 (54, 55), etc., ultimately promoting the expression of a series of genes that coordinate and regulate endochondral osteogenesis.

*In vitro* experiments have shown that transplanting chondrocytes and subchondral bone fragments into bovine cartilage significantly improves survival compared to transplanting chondrocytes onto bovine cartilage alone (56). After co-culture of chondrocytes with osteoblasts from the subchondral osteosclerotic zone of humans, the gene expression of *SOX9*, *COL2*, parathyroid hormone-related peptide, and parathyroid hormone-related peptide receptor (PTH-R) in chondrocytes was significantly reduced. The gene expression of osteoblast-stimulating factor (OSF)-1 was elevated compared to those in osteoblasts derived from areas of subchondral osteosclerosis in humans (57). From the above studies, osteoblasts in different regions of the subchondral bone do enable differential expression of key factors in chondrocytes, more directly illustrating the interactive possibility of bone-cartilage crosstalk. In the osteoblast conditional knockout MMP13 animal model (58), osteoblast remodeling of the surrounding bone matrix is inhibited, resulting in reduced cartilage matrix proteoglycan content, reduced type II collagen, proteoglycan, and MMP13 production by chondrocytes, and increased incidence of cartilage lesions. Osteoblasts’ synthesis of MMP13 has an impact on the homeostasis of cartilage, proving that osteochondral interactions exist.

## 3 The biomechanical basis of exercise regulating bone-cartilage crosstalk in subchondral bone

### 3.1 Physiological structure

In the different stages of osteoarthritis development, prolonged inflammation leads to catabolism of bone and cartilage (59); meanwhile, hypoxia due to improper joint loading and pathological changes in the joint vasculature lead to damage bone and cartilage matrix (60, 61). And how osteoarthritis develops and worsens depends on the dynamic balance regulated by all these changes in the subchondral bone microenvironment. This dynamic balance is primarily governed by interactions between bone and cartilage cell types from a cellular and tissue perspective. There is now substantial evidence indicating that the development and progression of arthritis are accompanied by altered survival rates of osteoblasts, osteocytes in bone, as well as chondrocytes in cartilage. Degeneration of cartilage tissue is a hallmark feature of arthritis. Numerous studies have now shown that the entire joint tissue of the body is involved in the process of arthritis. Crosstalk between cartilage and subchondral bone is thought to be the primary characteristic of this process.

Physiological structure from an anatomical perspective, the articular cartilage overlies the subchondral bone and is in close contact. The conventional theory holds that the calcified layer of cartilage immediately above the subchondral plate and the subchondral plate act as an impenetrable barrier, which means it is impossible to achieve bone-cartilage crosstalk in structure. Yet a large body of evidence suggests that these tissues can communicate. Duncan (62) et al. described numerous small holes in the subchondral plate located below the area covered by the meniscus, some of which appear to penetrate the subchondral plate and connect to the bone marrow cavity. Later, to measure the solute transport in calcified cartilage in real time, Jun Pan et al. (63) created an imaging technique based on fluorescence loss due to photobleaching (FLIP) in 2009. They discovered that sodium fluorescein could penetrate from subchondral bone into calcified cartilage. These findings suggest that there may be direct signaling between subchondral bone and articular cartilage, which form functional units with mechanical and biochemical interactions that may play a role in the maintenance and degeneration of the joint (63). This not only confirms the findings of Duncan et al., but also aligns with the 2006 discovery that human cartilage osseointegration is more intricate than previously thought: uncalcified cartilage often penetrates calcified cartilage and reaches into bone and bone marrow interstices (64). The results above are sufficient to indicate that there might be a molecular diffusion channel connecting the two spacers, and this idea has been empirically supported. On the other hand, novel OA therapeutic modalities based on bone-cartilage crosstalk between subchondral bone and cartilage have emerged. Bisphosphonates (BPs) are used as efficient bone-targeting agents to inhibit osteoclast activity, and biomaterials coupled to BPs are also used to carry other active molecules for basic bone-related therapies (65). BPs-modified nanoapatite (NP-BP) enables the targeting of subchondral bone by responding to lower pH in the microenvironment (65), and this system overcomes the deficiency of passive defense against bone resorption by BPs. It can effectively inhibit osteoclastic bone resorption. They showed that the injectable NP-BP system could inhibit abnormal subchondral bone remodeling, abnormal angiogenesis and excessive upward invasion of subchondral bone into calcified cartilage, thereby attenuating cartilage degeneration by inhibiting overactive osteoclast activity in a rat model of osteoarthritis (65). It is believed that the injectable NP-BP system has potential applications in osteoarthritis and other osteoarticular diseases. In addition, targeting crystalline mineral loss and reducing collagen mineralization in subchondral bone may also be potential targets for OA (66). Due to the natural barrier of articular cartilage and low blood circulation in the subchondral bone, there are tremendous difficulties in targeting subchondral bone for treatment options. Although research targeting subchondral bone is limited, the future development of subchondral bone-targeting biomaterials based on surface modification and innovative structures will be a new direction for OA treatment (67).

### 3.2 The correlation between osteoporosis and osteoarthritis is the embodiment of bone-cartilage crosstalk

Based on the above structural basis, it is evident that a strong linkage exists between osteogenesis and cartilage degeneration during the progression of osteoarthritis. The most typical manifestation is the correlation between osteoporosis and osteoarthritis. Osteoporosis is an age-related systemic metabolic disease with reduced bone mass and bone density as the main pathological changes and is most common in postmenopausal women. Knee OA is also a common and prevalent disease in postmenopausal women, and the prevalence of Knee OA is climbing as the aging of the Chinese population becomes more and more serious (11). OA and osteoporosis (OP) are two different diseases, and multiple factors can influence the development of both. According to existing studies, age, gender, genetics, chronic inflammation, endocrine, and metabolism are considered common risk factors for both diseases, while body mass index (BMI), BMD, and mechanical loading of joints may play different roles in the development of both diseases (68). Although several studies have been conducted on bone density levels in the lumbar spine and hip of patients with osteoarthritis and concluded an association between osteoarthritis and osteoporosis, there is still much controversy about the relationship between the two diseases and their mutual effects. We believe that changes in bone mass during osteoporosis correlate with osteoarthritis as a macroscopic manifestation of cartilage-to-bone crosstalk. Bone mass can laterally reflect the level of bone strength, and BMD tests can show the changes in bone mass more accurately, and there are early studies related to BMD in both OA and OP in pathological states. Nevitt et al. (69) conducted a study on the relationship between hip osteoarthritis and bone density using radiographic imaging. Through linear regression analysis, they discovered that patients with moderate to severe OA had higher bone density in the proximal femur, spine, and extremities compared to those with no or mild OA features. The authors attributed this phenomenon to secondary bone remodeling caused by hip OA in the proximal femur. Jan Dequeker (70) speculated that OA and OP might be linked through bone mass and that patients with OA may delay or hinder the onset of OP because they exhibit higher bone mass. Zhang Y et al. (71, 72) found that high BMD levels delayed the progression of knee OA but did not prevent its occurrence, and the protection of the knee joint by high BMD may be mainly related to the protection of the joint space. Some scholars even discovered that pain in KOA patients might be related to bone loss (73). The aforementioned research is adequate to demonstrate the link between osteoporosis and osteoarthritis.

### 3.3 Exercise enhances the mechanical crosstalk between bone and cartilage in the subchondral bone microenvironment

Regular exercise is one of the most effective measures to prevent osteoporosis (74), and high-intensity progressive resistance and impact training has the extraordinary ability to improve bone mass and function in older men and women and thus to reduce fracture risk (75–77). Mechanical signaling helps prevent bone loss and reduce the risk of fracture, even if it does not stimulate an increase in bone mass (78). Running is a common form of mechanically loaded exercise, and low-intensity running maintains cartilage homeostasis (79). The skeletal system serves as the mechanical skeleton of the entire body, and increased mechanical loading can treat postmenopausal osteoporosis (11). And the decrease in knee joint loading results in a significant decrease in subchondral bone mass and a reduction in articular cartilage layer thickness in mice, suggesting that appropriate joint loading plays a vital role in maintaining the homeostasis of articular cartilage and subchondral bone. Therefore, we believe that exercise-induced mechanical loading also acts as a catalyst in addition to strengthening the bond of bone-cartilage crosstalk.

We believe that describing the impact of exercise on subchondral bone-cartilage crosstalk in osteoarthritis should consider both the intensity of exercise and the course of osteoarthritis development in a thorough manner. In the study of exercise for osteoarthritis, treadmill running is widely used as an exercise intervention for modeling mice with osteoarthritis. The mechanical overload generated by running is an important factor in the development of osteoarthritis. The different effects of different intensities of treadmill running exercise on osteoarthritis in murine can be understood as a response of the cartilage and subchondral bone microenvironment to mechanical stresses of different loading intensities. In animal experiments, the actual exercise intensity can be controlled by measuring the maximum oxygen uptake to develop the exercise protocol for the experiment (80). Moderate-intensity exercise corresponds to approximately 50% to 70% VO_2_ max, while treadmill running exercise intensities greater than 70% VO_2_ max are considered high-intensity exercise (81). Of course, in the case of rats, the modeling of osteoarthritis is not limited to the treadmill running alone. However, it can be combined with other modalities as well. Coyle et al. (79) attempted to create an OA rat model using the anterior cruciate ligament transectionthree weeks after surgery plus treadmill running exercise. The histological Mankin score and anabolic indexes of articular cartilage in the experimental group showed successful OA modeling. Postoperative knee surgery in rats leads to increased articular cartilage wear, and subsequent running exercise aggravates articular cartilage damage, which can advance the OA lesion process, thereby shortening the experimental modeling period or causing lesions in the middle and late stages of OA.

Ni et al. (82) induced OA in rats using high-intensity treadmill running exercise at >70% VO_2_ max and joint braking simultaneously. The findings revealed that both the high-intensity runner exercise group and the joint braking group had significantly lower levels of extracellular matrix anabolic-related proteins like joint proteoglycan and type II collagen and that the articular cartilage damage in the braking group was more severe than in the high-intensity exercise group. According to Yao Z. et al., strenuous running inhibits PDGF-AA synthesis in the subchondral bone. It leads to the downregulation of PDGF/Akt signaling in articular cartilage, resulting in cartilage degeneration (83). Excessive mechanical stress can induce mitochondrial DNA damage and mitochondrial dysfunction by modulating the p53R2/p53AIP1 protein to activate the mitochondrial apoptotic pathway, which ultimately leads to chondrocyte apoptosis (84). This implies that high-intensity running exercise caused early OA while joint braking exercise caused mid to late-stage OA to form. Compared to high-intensity treadmill running exercises, low-medium-intensity treadmill running exercises are mostly used in experimental animal studies on treating osteoarthritis in murine. Regular exercise training for four weeks alleviates cartilage degeneration in model rats with KOA (85). It has been observed that mild and short-term treadmill walking can safeguard chondrocytes in a rat model by preventing an upsurge in osteocyte mortality (86). Treadmill training over four weeks alleviates subchondral bone loss and remodeling and reprograms the cartilage-subchondral unit (87). The expression of matrix metalloproteinases (MMPs) in articular cartilage depends on the intensity of mechanical stress stimulation. Moderate-intensity exercise inhibits the expression of MMPs, thus reducing apoptosis and extracellular matrix degradation in particular chondrocytes (88). Mechanism studies in exercise treatment of murine osteoarthritis focus mostly on the regulation of key factors and signaling pathways by exercise. Aerobic exercise reduced expression in IL-1β, cystein-3 and MMP-13 and prevented KOA-induced cartilage degeneration in model rats (89). Running machine and wheeled exercise reduced the levels of IL-1β, IL-6 and TNF-α and modulated JNK/NF-κB signaling to prevent inflammation in a model rat with KOA (90). Moderate physical exercise prevents B-type synovial cell dysfunction and delays disease progression in rats with early osteoarthritis (91).In addition, the selection of exercise periods for the treatment for rodent osteoarthritis is also very important.An animal study showed that early intervention with swimming was more effective than delayed intervention in the early stages of cartilage injury when post-traumatic osteoarthritis had already developed (92). Further evidence supporting the role of exercise in regulating bone-cartilage crosstalk in osteoarthritis, involving significant factors and signaling pathways, will be presented subsequently.

### 3.4 The role of exercise therapy in arthritis rehabilitation

As a non-pharmacological therapy, exercise therapy is currently employed extensively in treating osteoarthritis. One of the prerequisites for preserving the stability of the skeletal system is mechanical stress stimulation, which is produced on the skeleton during movement of the body by gravity, ground reaction forces, and skeletal muscle contraction (93). Scientific exercise interventions have been shown to improve proprioception, increase periarticular muscle strength, and restore the biomechanical balance of periarticular tissues (94). Exercise therapy is an efficient way to treat knee pain and dysfunction in KOA patients, and it is a treatment that is both safe without side effects and easily accepted by patients (95). In recent years, various human studies have investigated the efficacy of different exercise interventions on osteoarthritis, such as plyometric training, aquatic exercise, aerobic exercise, neuromuscular motor control training, balance training, proprioceptive training, and traditional exercise. And the mechanism of exercise for osteoarthritis is discussed from the biomechanical perspective (12, 94, 96, 97).

**Figure 1:**
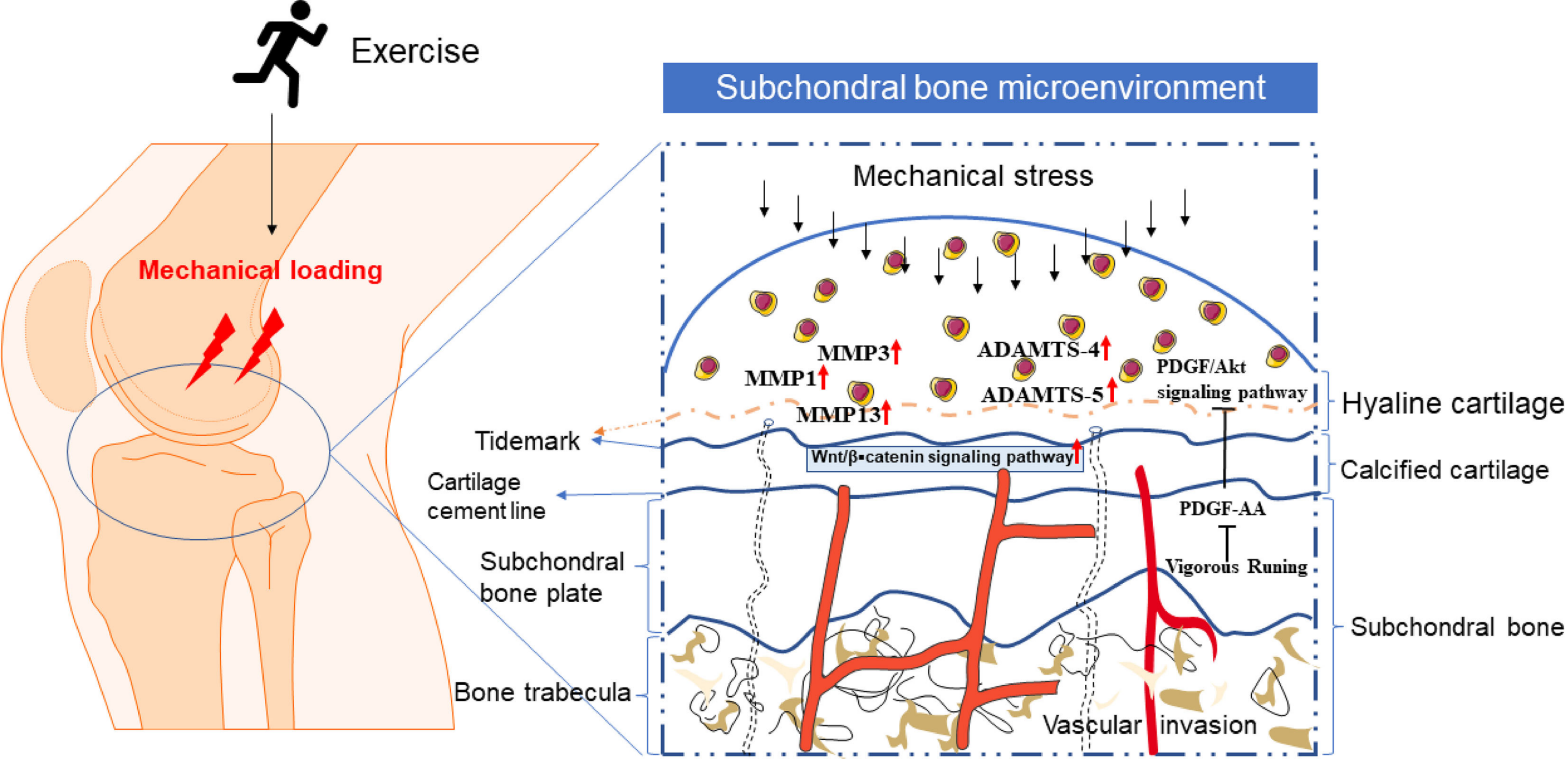
The microenvironment of cartilage and subchondral bone under exercise or mechanical stress. Mechanical loading caused by exercise or other factors can penetrate the scope of articular cartilage. Proper exercise will not harm normal articular cartilage. However, when the lesion of normal cartilage or arthritis aggravates, the tidal line under hyaline cartilage moves upward (the tidal line represented by the dotted line), the calcification of articular cartilage increases, and the articular cartilage degenerates. The pathway of bone-cartilage crosstalk in subchondral bone, such as vascular invasion and holes that can pass through the subchondral plate into the bone marrow, may be the basis and ways for exercise to regulate bone-cartilage crosstalk.

As a common form of mechanically loaded exercise, and the intensity of the exercise plays a crucial role in the development of osteoarthritis, treadmill running is often used for experimental animal modeling. The growth and maintenance of knee ligaments, bones, and cartilage are all encouraged by adequate exercise intensity (98), which also improves muscle strength around the joint to lessen the strain on the joint suitably. However, vigorous platform running may put a more mechanical strain on the knee, which could lead to articular cartilage degradation (7). Low-intensity mechanical stress inhibits the expression of inflammatory genes in articular cartilage and decreases joint pain, according to related ex vivo investigations (99). On the other hand, excessive mechanical stress is known to encourage the development of inflammatory factors in articular cartilage and can damage the cartilage matrix. Therefore, moderate exercise can effectively relieve joint pain by enhancing the biomechanical relationship around the joints, controlling the expression of inflammatory factors in articular cartilage, and significantly impacting the treatment and prevention of osteoarthritis (**Figure 1**).

## 4 A molecular mechanism for the exercise regulation of bone-cartilage crosstalk

The observations we have summarized above indicate the likelihood of direct signaling transmission between subchondral bone and articular cartilage, suggesting that cartilage and bone form a functional unit mechanically and biochemically, which may play an essential role in intra-articular homeostasis and disease. A number of cytokines and growth factors may have a role in the high-frequency alteration of subchondral bone during the course of arthritis. According to the review by Lajeunesse in 2004 (100), these cytokines and growth factors can penetrate the cartilage that covers the surface of subchondral bone and control chondrocyte biology, thus establishing a positive feedback loop between cartilage and subchondral bone.

### 4.1 Signaling pathways involved in the exercise-regulated bone-cartilage crosstalk

**Figure 2:**
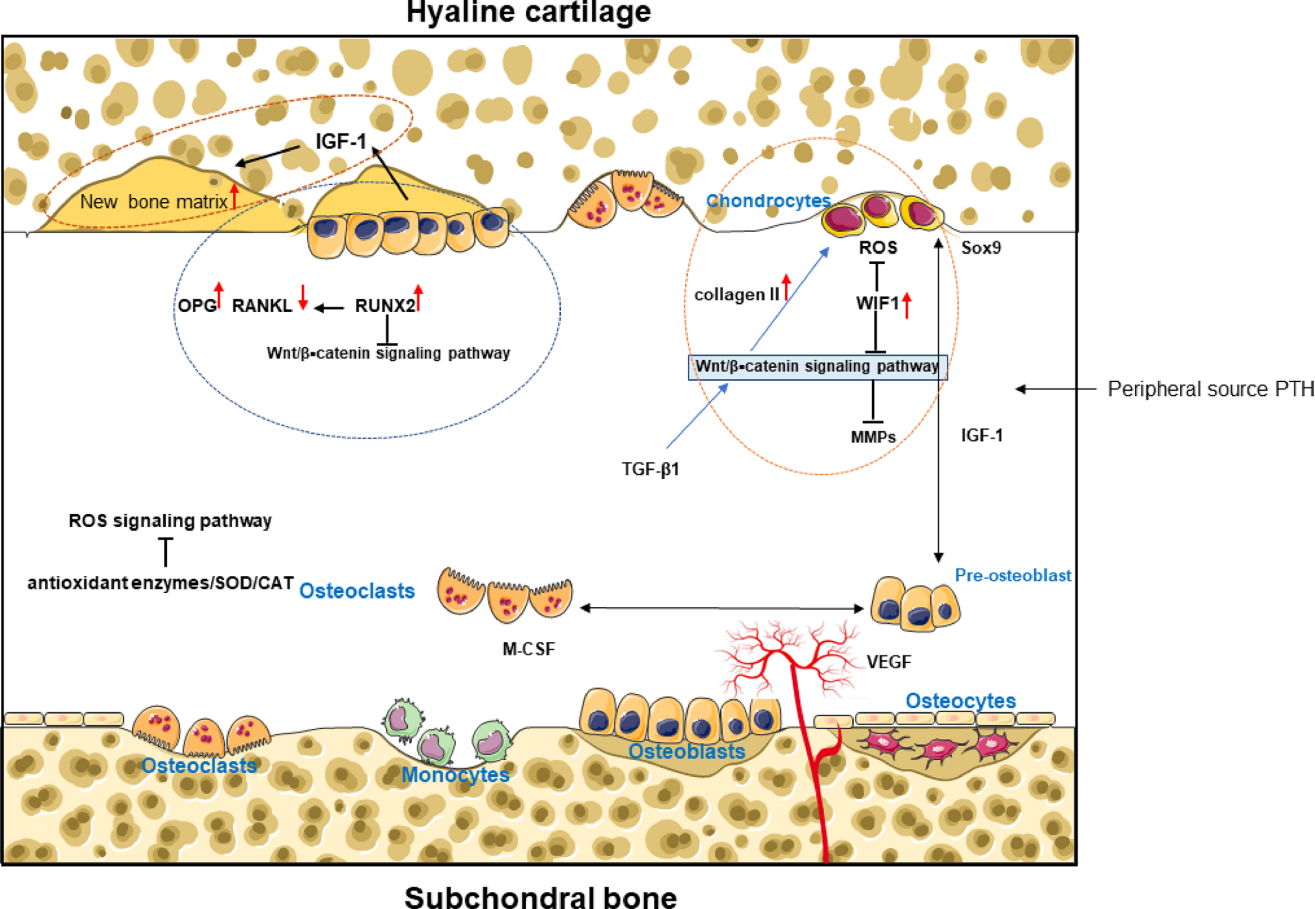
Cytokines and growth factors regulate chondrocyte biology and establish a positive feedback loop via biochemical crosstalk between cartilage and subchondral bone. In the cartilage and subchondral bone microenvironment, osteoblast-dominated bone formation and osteoclast-dominated bone resorption affect cartilage formation or cartilage degeneration. (**Figure 2**) mainly shows the main signal pathways and cytokines of bone-cartilage crosstalk in the subchondral bone under exercise intervention.

Many studies have shown the presence of abnormal subchondral bone remodeling in the progression of osteoarthritis (101, 102). This remodeling process is dominated by chondrocytes, osteoblasts, osteocytes, and mesenchymal stem cells, and bone remodeling in the course of OA includes bone formation and bone resorption. It is now generally accepted that subchondral bone exhibits bone resorption in the early stages of OA and excessive bone sclerosis in the late stages of OA. A large number of changes occur in the behavior of cartilage and bone cells, which leads to changes in the expression of many molecules that may have both an autocrine role in their producing tissues and may contribute to altering the dialogue between bone and cartilage, as proteins and other larger molecules are able to be transported between these tissues. In the case of chondrocytes (103),osteoblasts and osteocytes (104, 105) both have good mechanosensing capabilities, especially in the overload state. Mechanical stress stimulation plays an important role in cartilage-bone crosstalk Mechanical stress stimulation is able to facilitate information exchange between tissues or cells to some extent. Many signaling pathways, including Wnt/β–catenin, RANK/RANKL/OPG, and ROS involved in both chondrogenesis and osteogenesis, are also sensitive to mechanical stress stimulation (**Figure 2**).

The Wnt/β–catenin signaling pathway is a more typical representative. β-catenin is a multifunctional protein in the cytoplasm and a key molecule in the Wnt signaling pathway that regulates gene transcription (106). The Wnt/β-catenin signaling pathway is a key regulator of bone, cartilage, joint development, and homeostasis. It plays an important role in many biological processes, including the cohesion and differentiation of mesenchymal cells, maintenance of the phenotype of mature articular cartilage, and maturation of hypertrophic tissue during endochondral osteogenesis (107). This is the basis for the Wnt/β-catenin signaling pathway that maintains the cartilage and subchondral bone endoskeletal environment homeostasis.Most importantly, it plays an essential role in regulating the function of osteoblasts, osteoclasts, and osteocytes and influences skeletal development and homeostasis. Abnormal Wnt signaling can lead to diseases such as skeletal defects (108). Upregulation of Wnt inhibitory factor 1 (WIF1) would promote OA chondrocyte proliferation, inhibit apoptosis by eliminating ROS production, and reduce MMP secretion by blocking the Wnt/β-linked protein signaling pathway (109). Adequate mechanical loading promotes bone formation and increases SB thickness and trabecular volume fraction by activating Wnt/β-linked protein signaling. Related studies have shown that upregulation of β-linked protein and Wnt-3a was found in both the injury exercise-induced OA model group and the exercise-induced OA model group (110). Thus, β-linked protein and Wnt-3a may be involved in the pathogenesis Page 7of exercise-induced OA through the abnormal activation of the Wnt/β-linked protein pathway by frequent excessive stress during exercise.

The OPG/RANKL/RANK signaling pathway is an important signaling channel for the interaction between osteoblasts and osteoclasts. Among them, RANKL and OPG are considered to be key molecules in regulating bone reconstruction. Both factors are produced by osteoblasts/stromal cells as well as human chondrocytes, whereas the RANK receptor is only expressed in human osteoarthritic chondrocytes (111). Compared to normal cartilage, the RANKL/OPG ratio is increased in both human osteoarthritic cartilage and in mandibular osteoarthritic synovial cartilage from osteoarthritic rats (111, 112). Overall, the RANKL/OPG ratio increases and then decreases in OA (113), consistent with the pathological changes in OA where early bone resorption predominates and late bone formation predominates.The RANK/RANKL/OPG signaling pathway regulates subchondral bone remodeling through the Wnt/β-linked protein signaling pathway. On the one hand, the OPG/RANKL signaling pathway is involved in the secretion of β-linked proteins by chondrocytes (114). However, in osteoblasts,OPG expression is regulated by the Wnt/β-linked protein signaling pathway (115). On the other hand, the knockdown of Runx2, a key regulator of bone formation, induces high expression of RANKL and suppresses OPG expression. In contrast, overexpression of Runx2 inhibits the function of the typical Wnt/β-linked protein signaling pathway by depleting β-linked protein, resulting in reduced bone volume and volume (116). However, due to Runx2 overexpression, β-linked protein activation reverses the high bone resorption in mouse subchondral bone, which is closely associated with RANKL/OPG signaling (116).

Inflammatory factors are closely related to stress itself;inflammatory factor expression induces chondrocyte stress, and prolonged stress induces inflammatory factor secretion. During stress, a large amount of active ROS is produced, eventually leading to chondrocyte damage if the excess ROS is not cleared in time (117). Exercise itself is also a kind of stress, as oxygen consumption increases during exercise, and a large amount of ROS is produced to meet the body’s oxygen demand, at which time the activity of antioxidant enzymes is also enhanced to improve the body’s tolerance to oxidative stress and remove excess ROS. Generally, moderate aerobic exercise can significantly increase the activity of the antioxidant enzymes such as superoxide dismutase (SOD). Kaczor et al. (118) found that long-term low-intensity exercise increased adaptive cellular responses, slowed cellular damage caused by ROS, and inhibited apoptosis. We suggest that the Wnt/β-linked protein signaling pathway and OPG/RANKL/RANK signaling pathway are highly activated when articular cartilage is damaged. In contrast, appropriate exercise or mechanical stress stimulation inhibits the activation of these pathways and gradually stabilizes them by reducing inflammatory factors and chondrocyte stress.Low to moderate-intensity aerobic exercise can be an excellent way to intervene in damaged cartilage.

### 4.2 Cytokines involved in the exercise regulation of bone-cartilage crosstalk

IGF is one of the important growth factors that regulate bone formation. In 2005, Koch (119) et al.found that IGF can up-regulate the expression of early osteogenic genes in human bone marrow mesenchymal stem cells (hBMSCs), including type I collagen, alkaline phosphatase, and Runx2, and thus to promote the osteogenesis of hBMSCs. In osteoarthritis, osteoblasts of the subchondral bone can produce large amounts of different types of IGF-1, while production of IGF-1 binding protein is reduced compared to normal subchondral bone (120), so large amounts of free IGF-1 promote bone reconstruction and lead to the development of osteosclerosis, which simultaneously exacerbates cartilage matrix degradation. Studies have shown that 12 weeks of combined anaerobic and aerobic exercise improves IGF-1 levels as well as insulin resistance in older women and that exercise can affect IGF-1 levels (121). Matheny et al. (122) found increased locomotor performance, up-regulated IGF-1 mRNA expression in muscle, and increased muscle weight in the hind legs of adult liver IGF-1 conditional knockout mice after 16 weeks of ladder climbing endurance training. Currently, although there are fewer studies on exercise regulation of IGF-1 expression in relation to osteoarthritis, it is not difficult to make the hypothesis that exercise can be involved in regulating bone-cartilage crosstalk in subchondral bone through IGF-1 based on its important interaction in osteogenesis and osteoarthritis, and its sensitivity to exercise performance.

Transforming growth factor β1 (TGF-β1) is a cytokine that plays an important role in the induction of chondrogenesis (123). TGF-β1 expression is significantly higher in healthy cartilage than in OA cartilage (124). In the progression of chondrocyte phenotypic degeneration including senescence and dedifferentiation, downregulation of TGF-β1 indirectly induces disorders of chondrocyte metabolism (125). TGF-β1 promotes chondrocyte proliferation through β-linked protein signaling and maintains the chondrocyte phenotype by enhancing collagen II synthesis (125, 126), suggesting that TGF-β acts as a mediator for cartilage and subchondral bone (127). Zhen et al. (128) studied the rat OA model with anterior cruciate ligament ostomy. They found that TGF-β in subchondral bone activated responsively to altered mechanical loading. Increased TGF-β in subchondral bone increased the number of mesenchymal stem cells, osteoprogenitor cells, and osteoblasts, leading to abnormal bone reconstruction as well as angiogenesis. It is important evidence for bone-cartilage crosstalk under mechanical stress stimulation conditions. In addition, R. K. Zhang (129) et al. also conducted a related study on the response of TGF-β1 signaling pathway to mechanical overload in osteoblast and chondrocyte co-cultured cells, and the results showed that mechanical stress might be a trigger for TGF-β1 upregulation in osteoblasts.

Mechanical stress intervention in osteoclasts inhibits chondrocyte proliferation and can induce apoptosis in the osteoclast/chondrocyte co-culture system (129). Transgenic expression of active TGF-β1 in osteoblasts is sufficient to induce osteoarthritis. In contrast, direct inhibition of TGF-β1 activity in subchondral bone attenuates degeneration of articular cartilage (123), suggesting that exercise, or exercise-induced mechanical stress, plays an important role in the cartilage-osteoblast-broken bone-dominated subchondral bone transition and bone homeostasis. This further confirms the role of bone-cartilage crosstalk catalysis by mechanical stress in the subchondral bone microenvironment. Of course, this catalytic effect cannot be achieved without the participation of various cytokines, and here we only describe IGF and TGF-β1 in detail. Other factors playing messenger roles are Sox9, PTH, M-CSF, VEGF, etc. Among them, macrophage colony-stimulating factor (M-CSF), although indispensable in the proliferation and differentiation of osteoblasts as well as in the fusion of their cellular precursors, also regulates the resorptive activity of mature osteoblasts, and its dissemination in the cytoplasm simultaneously inhibits the apoptosis of mature osteoblasts (130), but it is not associated with a strong sensitivity to mechanical loading.

Sox9 plays a key role in the differentiation of mesenchymal cells into chondrocytes (131), and Sox9 can be found in abundance at the site of chondrogenesis, as well as when mesenchymal cells are concentrated prior to differentiation into chondrocytes. Invitro studies conducted on cultured chondroprogenitor cells have demonstrated that biomechanical stimulation significantly promotes the differentiation of chondroprogenitor cells and the growth of the extracellular matrix. Moreover, the Sox9 gene has been found to play a crucial role in this process, which suggests its importance in stress transmission and response (132). At present, there are many kinds of exercise interventions in animal studies related to exercise-mediated Sox9 treatment of osteoarthritis, for example, treadmill running, Combined therapies with exercise, ozone, and mesenchymal stem cells (133), vibration exercise with different frequencies (134).

Parathyroid hormone (PTH), the most important regulator of calcium and phosphorus metabolism in humans, increases reactively when serum calcium levels decrease. And its overproduction leads to the development of bone resorption, while low, intermittent doses act to promote increased bone mass (135). After stimulating the skeleton with dynamic loading, exercise results in a systemic rise in parathyroid hormone (PTH) (136). Both clinical and animal studies have found a transient release of PTH in nature by following a single exercise session (137–140). In addition to PTH released by the parathyroid glands, exercise causes local expression of PTH-related peptide (PTHrP) and PTH/PTHrP type 1 receptor (PPR) (141, 142), while exercise significantly increases PTHrP production by osteoblasts, this then causes PPR to get activated in an autocrine or paracrine way. Recent animal experiments have shown that the synthetic parathyroid hormone PTH (1–34) improves the structure of cartilage surfaces in OA and contributes to subchondral bone reconstruction (143). PTH signaling has a unique role in bone adaptation during exercise, which is mediated through the activation of PTH-related peptide type 1 receptor (PPR) along the osteoblast spectrum (144). In 2014, Yan et al. (145) found that PTH (1–34)-treated cartilage had increased type II collagen expression, decreased SOST expression, an increased OPG/RANKL ratio, and an increased amount of subchondral trabecular bone compared to controls. It was shown that PTH (1–34) has a role in preventing OA cartilage destruction and delaying subchondral trabecular bone degeneration. PTH and its associated receptors are sensitive to exercise load, and the involvement of PTH in the bone-cartilage crosstalk of subchondral bone has been well-documented in related studies. It is likely that PTH is a potentially critical factor in the exercise-promoted bone-cartilage crosstalk of subchondral bone. However, the specific mechanism by which exercise modulates the subchondral bone-cartilage crosstalk via PTH needs to be investigated in more depth.

## 5 Summary

The review summarizes the feasibility of exercise to promote bone-cartilage crosstalk in subchondral bone and the related molecular mechanisms. Excessive mechanical stimulation triggers cartilage lesions,and moderate exercise promotes cartilage repair.This suggests differences in the effects of different intensities of exercise on the bone-cartilage crosstalk of subchondral bone.Bone-cartilage crosstalk is the basis for maintaining subchondral bone transition and bone homeostasis. Exercise promotes the effect of bone-cartilage crosstalk mainly by regulating mechanosensitive cytokines and signaling pathways such as Wnt/β⁃catenin, RANK/RANKL/OPG, and ROS signaling pathways. However, many questions still need to be answered in more scientific and comprehensive related studies. For example, do relevant animal and *in vitro* studies provide valuable information on specific exercise protocols for exercise prevention and treatment of osteoarthritis in humans? Indeed, with the advancement of high-resolution tools such as MRI, we will be able to explain the positive modulatory effects produced by exercise more visually. At the same time, the creation of new responsive biomaterials targeting subchondral bone opens up more possibilities for the treatment of osteoarthritis patients. Thus, in the future, the combination of exercise and nascent therapeutic modalities is a new potential way whereby exercise prevents and treats osteoarthritis.

## Author contributions

SZ: conceptualization, formal analysis, resources, writing-original draft, writing-review and editing. TL: writing-original draft, writing-review and editing. YF: data curation, formal analysis, and editing. KZ: data curation, writing-review and editing. JZ: writing-review and editing. XW: writing-review and editing.YY: funding acquisition, supervision, writing-original draft, writing-review and editing. LZ: funding acquisition, supervision, writing-review and editing, project administration. All authors contributed to the article and approved the submitted version.
